# Tumor Budding Score Is a Strong and Independent Prognostic Factor in Patients With Pancreatic Ductal Adenocarcinoma: An Evaluation of Whole Slide Pathology Images of Large Sections

**DOI:** 10.3389/fonc.2021.740212

**Published:** 2021-11-30

**Authors:** Hui Jiang, Yelin Yang, Yuping Qian, Chengwei Shao, Jianping Lu, Yun Bian, Jianming Zheng

**Affiliations:** ^1^ Department of Pathology, Changhai Hospital, The Naval Military Medical University, Shanghai, China; ^2^ Department of Radiology, Changhai Hospital, Naval Medical University, Shanghai, China

**Keywords:** pancreatic neoplasm, carcinoma, tumor budding, prognosis, survival, neoplasm staging

## Abstract

**Objective:**

We aimed to develop the tumor budding (TB) score and to explore the association between the TB score and overall survival (OS) in patients with pancreatic ductal adenocarcinoma (PDAC).

**Methods:**

In this retrospective study, 130 consecutive patients with PDAC underwent surgical resection between July 2016 and March 2019. The location and counts of TB were assessed based on the digitalized whole slide hematoxylin and eosin images. The TB score was achieved using the Cox regression equation. The cutoff point for the TB score was determined by X-tile. Univariate and multivariate Cox regression models were used to analyze the association between the TB score and OS.

**Results:**

The TB score was 0.49 (range = 0–1.08), and the best cutoff for the TB score was 0.62. The duration of survival in individuals with a low TB score [median = 21.8 months, 95% confidence interval (CI) = 15.43–25.50] was significantly longer than that in those with a high TB score (median = 11.33 months, 95% CI = 9.8–14.22). Univariate analysis revealed that the TB score was significantly associated with OS [hazard ratio (HR) = 2.71, 95% CI = 1.48–4.96, *p* = 0.001]. Multivariate analysis revealed a strong and independent association between the TB score and OS (HR = 2.35, 95% CI = 1.27–4.33, *p* = 0.03). The high TB score group had a 2.14 times higher mortality than the low TB score group.

**Conclusion:**

The TB score is strongly and independently associated with the risk of OS in PDAC.

## Introduction

Pancreatic ductal adenocarcinoma (PDAC) is a highly fatal malignancy with a 5-year survival rate of only 9% and was the seventh leading cause of cancer-related death in both sexes worldwide in 2018 (1). Surgery offers the only probable opportunity for cure. Unfortunately, recurrence is observed even in patients who have undergone complete resection and have node-negative PDAC (2). Difficulty in early detection and non-response to treatment have led to high mortality in patients with PDAC (2). Therefore, there is a need for reproducible and reliable prognostic markers that would enable better patient stratification and eventually provide a guide for more successful and individualized therapy.

Tumor budding (TB), defined as the presence of isolated single cancer cells or clusters of up to four cancer cells at the invasive tumor front, has emerged as a potential prognostic biomarker in some solid tumors, predicting disease progression and adverse survival (3). Biologically, TB is associated with epithelial–mesenchymal transition (EMT) in cancer (4). EMT is a biological process that leads to enhanced cell migration, invasiveness, and increased resistance to apoptosis (5). TB was first proposed for colorectal cancer and has been recognized as a marker of aggressiveness or adverse prognostic factor of events (6, 7). This histological parameter has been demonstrated as an independent prognostic marker in other cancer types (8–11). It has been reported that TB in patients with PDAC has a clear association with the process of EMT and adverse prognosis (12–17). Previous studies usually focused on “classic” peritumoral budding (PTB). Furthermore, TB can also be found within the main tumor body in colorectal cancer; therefore, the term “intratumoral budding” (ITB) has been introduced to distinguish this form of budding from the “classic” PTB (18–20). However, ITB in PDAC is rarely reported (15), and there is nearly no studies combining the count and location of TB. Here, we aimed to develop a TB score combining the budding count and location based on whole slide pathology images of large sections and to further explore the association between the TB score and overall survival (OS) in patients with PDAC.

## Methods

### Patients

**Figure 1:**
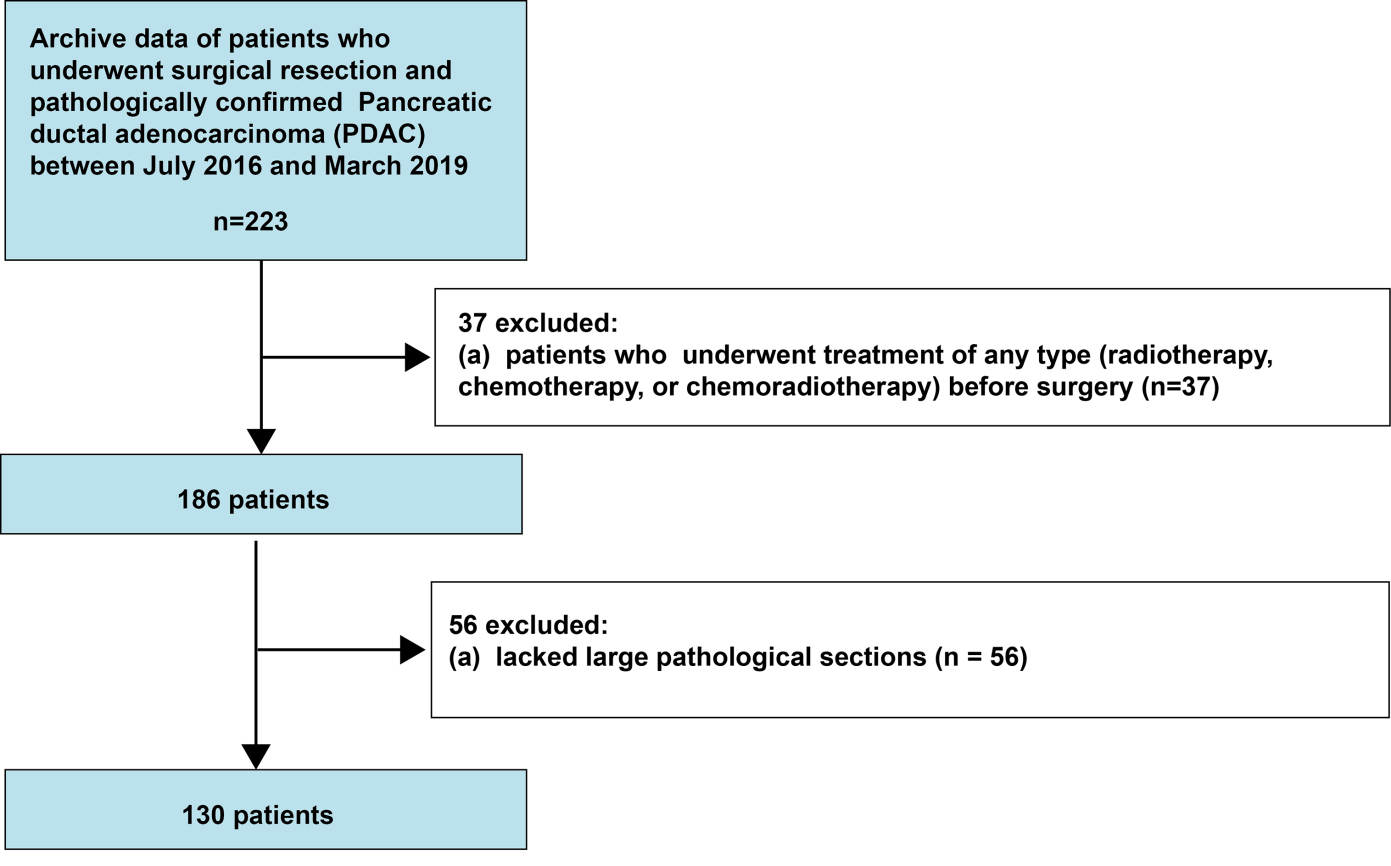
Flowchart indicating the patient selection process.

This retrospective, single-center, cross-sectional study was reviewed and approved by the Biomedical Research Ethics Committee of our institution (no. CHEC-Y2015-011). The need for informed consent was waived by the Institutional Review Board. The clinical and histopathological data of consecutive patients who were treated for pancreatic cancer at our institution between July 2016 and March 2019 were retrieved (**Figure 1**).

We included patients who had undergone surgical treatment and had pathologically confirmed PDAC. We excluded patients who underwent any treatment (radiotherapy, chemotherapy, or chemoradiotherapy) before surgery and for whom large pathological sections were not available. Ultimately, 130 consecutive patients with PDAC were included.

### Pathological Image Analysis

All specimens were analyzed by two pathologists (HJ and YY, with experience of 20 and 10 years, respectively) who were blinded to the clinical data of the patients. The results were determined by consensus. A standard protocol for pathological examination and analysis, as described previously (21), was followed. The resected specimens were immediately fixed in formalin for 24 h. Subsequently, they were cut horizontally into 5-mm tissue blocks (slicing of the pancreaticoduodenectomy specimen in the axial plane and slicing of the distal pancreatectomy specimen in the plane perpendicular to the main pancreatic duct) that were dehydrated and embedded in paraffin; all pancreatic tissue was completely sampled. Finally, 5-μm-large sections (area = 76 mm × 52 mm) were prepared and stained with hematoxylin and eosin (HE). We aimed to include the tumor area in a large section. Each large section was converted to digital pathological images using a scanner (NanoZoomer S60; Hamamatsu Healthcare, Hamamatsu, Japan).

The following histopathological parameters were analyzed: 1) T and N categories, which were evaluated on the basis of the American Joint Committee on Cancer TNM Staging Manual, 8th edition (22); 2) grade of differentiation; 3) peripancreatic fat invasion; and 4) resection margin.

### Evaluation of TB

**Figure 2:**
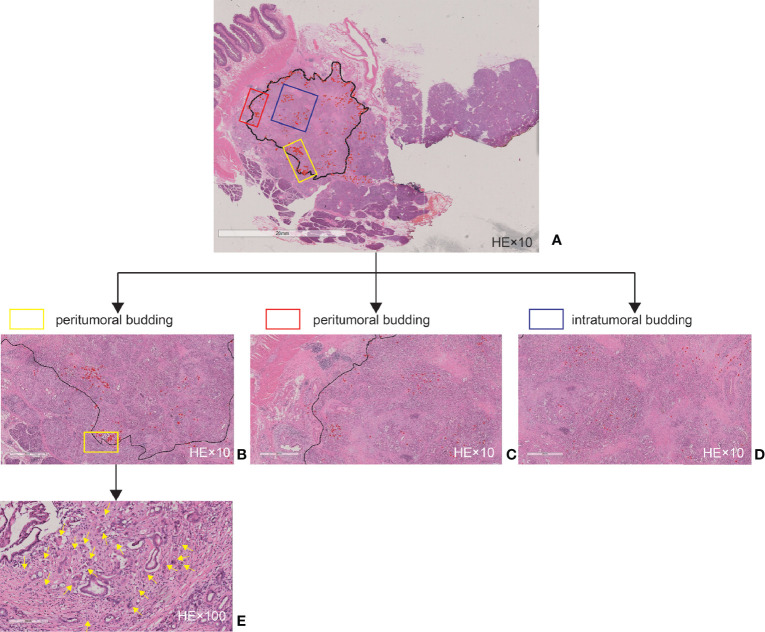
Tumor budding as assessed by large sections. **(A)** Example of a whole slide image. **(B)** Magnified high-power field (HPF, ×10) area of **(A)** at the tumor front. **(C)** Magnified HPF (×10) area of **(A)** at the tumor front. **(D)** Magnified HPF (×10) area of **(A)** in the tumor center. **(E)** Magnified HPF (×100) area of **(B)**. The *black line* delineates the tumor invasive front, and the *red marks* and *yellow arrows* indicate the tumor budding.

Both the periphery and central areas of the tumor were considered because budding in both locations has been shown to be closely related in colorectal carcinoma (23). According to the recommendations of the International Tumor Budding Consensus Conference 2016 in colorectal cancer, TB was identified as a single tumor cell or a cell cluster of up to four tumor cells at the infiltrative front of the tumor on HE staining (23). To identify the densest area of budding (“hotspot”), each digital pathological image of the large sections was scanned at ×100 magnification. PTB, ITB, and total TB (TTB) were defined as TB at the tumor front, TB in the tumor center, and TB both at the tumor front and in the tumor center, respectively (18). Subsequently, the number of tumor buds in a high-power field (HPF, ×400) image in an area measuring 0.2 mm^2^ was counted with the Aperio ImageScope software v.12.3.2.8013 (Leica Biosystems, Nussloch, Germany). An HPF often refers to an area visible under the maximum magnification power of the objective lens (×40) being used. In our study, an HPF was considered as a field of 1,302 × 2356 pixels at ×400 magnification. Finally, we obtained the PTB and ITB counts (**Figure 2**).

### Statistical Analyses

Normal distribution and variance homogeneity tests were performed on all continuous variables using the Kolmogorov–Smirnov test. Those with a normal distribution are expressed as means and standard deviations, while those with non-normal distributions are expressed as medians and ranges. Interobserver agreement was quantified using the *k*-statistic for categorical variables (poor agreement: *k* = 0–0.20; fair agreement: *k* = 0.21–0.40; moderate agreement: *k* = 0.41–0.60; good agreement: *k* = 0.61–0.80; and excellent agreement: *k* = 0.81–1.00) and the intraclass correlation coefficient (ICC) for continuous variables (poor agreement: ICC = 0–0.49; moderate agreement: ICC = 0.50–0.75; good agreement: ICC = 0.76–0.90; and excellent agreement: ICC = 0.91–1.00) (24). If the agreements were good, we chose the results of HJ. We evaluated the OS; deaths were considered as events, and deaths attributed to other causes were considered censored observations. Survival times were calculated from the date of surgery to the time of death or the end of follow-up (August 1, 2020). Firstly, univariate Cox regression analysis was applied to estimate the effect size between all variables and OS. Secondly, the Cox regression model was used to construct a multi-budding feature-based classifier for predicting survival. The TB score was achieved using the Cox regression equation. The optimal cutoff TB score was determined with the help of X-tile (25). The X-tile program divided the patients into the high TB score group and the low TB score group based on the optimal cutoff value. Kaplan–Meier estimates were applied to plot the graph of the survival curves, and the log-rank test was performed to analyze the differences between the TB score-high group and the TB score-low group. Kaplan–Meier estimates and the log-rank test were also performed to analyze the differences between the absent TB group and the present TB group, between the TB count <5 and ≥5 groups, and between the TB count <10 and ≥10 groups. Thirdly, univariate regression analysis was also performed to estimate the effect size between all variables and the TB score. Finally, multivariable Cox regression models were used to evaluate the associations between exposure (budding location, TTB count, and TB score) and outcome (OS). These included: model 1 (not adjusted for other co-variants), model 2 (adjusted for age, sex, and body mass index), and model 3 (adjusted for the same factors as those in model 2 and other associated factors in the univariate regression analysis).

A two-tailed *p*-value less than 0.05 was considered statistically significant. All analyses were performed using R software (version 3.3.3; The R Foundation for Statistical Computing, Vienna, Austria).

## Results

### Clinical Characteristics

Interobserver agreements between two pathologists for the pathological characteristics were excellent, and the *k*-statistic ranged from 0.85 to 0.95. The interobserver ICCs of TB counts were also good, ranging from 0.87 to 0.93. A total of 130 consecutive patients with PDAC, 77 males (age = 61.61 ± 9.48 years, range = 40–82 years) and 53 females (age = 63.72 ± 7.00 years, range = 48–77 years), were included. Detailed patient and tumor characteristics are presented in **Table 1**.

**Table 1:** Results of the univariate analysis between all variables and overall survival.

Variable	Statistics	HR (95% CI)	*p-*value
Sex, *n* (%)			
Male	77 (59.23)	1.0	
Female	53 (40.77)	0.58 (0.37–0.91)	0.02
Age (years), mean ± SD	62.47 ± 8.59	1.00 (0.98–1.03)	0.79
BMI (kg/m^2^), mean ± SD	23.28 ± 9.36	1.01 (0.99–1.03)	0.37
Tumor size (cm) (median, range)	3.60 ± 1.47	0.95 (0.82–1.09)	0.46
Location, *n* (%)			
Head	76 (58.46)	1.0	
Body and tail	54 (41.54)	0.58 (0.37–0.92)	0.02
Grade of differentiation, *n* (%)			
Well–moderate	101 (77.69)	1.0	
Poor–undifferentiated	29 (22.31)	1.61 (0.97–2.67)	0.07
T category, *n* (%)			
T1	15 (11.54)	1.0	
T2	74 (56.92)	2.40 (1.03–5.60)	0.04
T3	41 (31.54)	1.59 (0.64–3.95)	0.32
N category, *n* (%)			
N0	42 (32.31)	1.0	
N1	40 (30.77)	1.23 (0.68–2.23)	0.49
N2	48 (36.92)	2.09 (1.22–3.61)	0.008
Peripancreatic fat invasion, *n* (%)			
No	45 (34.62)	1.0	
Yes	85 (65.38)	1.72 (1.07–2.77)	0.03
Resection margin, *n* (%)			
R0	72 (55.38)	1.0	
R1	58 (44.62)	1.24 (0.81–1.92)	0.32

HR, hazard ratio; CI, confidence interval; BMI, body mass index.

### Construction of the TB Score

**Figure 3:**
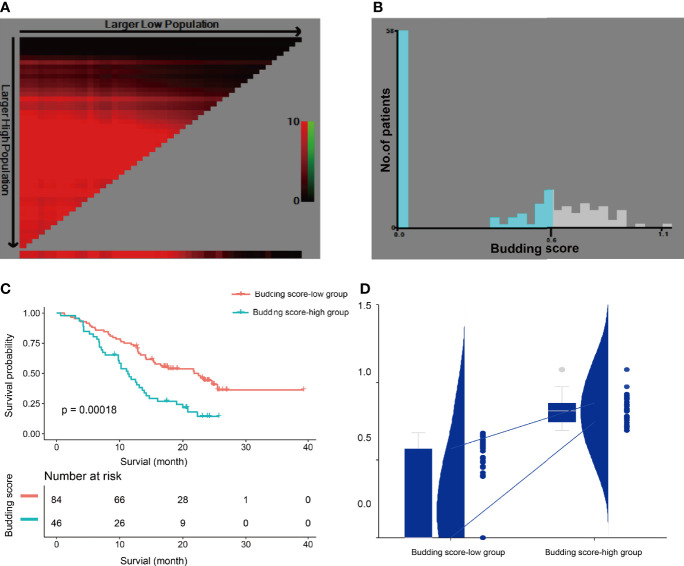
X-tile analysis of the survival data in patients with pancreatic ductal adenocarcinoma. **(A, B)** The optimal cutoff level of the tumor budding (TB) score is 0.62, which was determined by X-tile and used to define the TB score-low and TB score-high groups. **(C)** The Kaplan–Meier curve and log-rank test suggest that patients in the TB score-low group survived significantly longer than did those in the TB score-high group. **(D)** TB scores in the TB score-low and TB score-high groups. The chart includes a box plot, density plot, and a dot plot. The 25th and 75th percentiles are shown as *connecting lines* between the groups.

The TB score was achieved using the Cox regression equation (**Table 2**). Based on the optimal cutoff TB score level determined by X-tile (0.62) (**Figures 3A, B** **)**, all patients were divided into the TB score-low (TB score < 0.62, *n* = 84, 64.62%) and TB score-high (TB score ≥ 0.62, *n* = 46, 35.38%) groups. TB score expressions were 0 (range = 0–0.62) and 0.77 (range = 0.62–1.08) in the TB score-low and TB score-high groups, respectively. Fifty-four and 28 patients in the TB score-low and TB score-high groups died, respectively. The Kaplan–Meier curves of the two groups were significantly distinct (*p* = 0.002). A log-rank test showed that the survival duration in the TB score-low group [median = 21.8 months, 95% confidence interval (CI) = 15.43–25.50] was significantly longer than that in the TB score-high group (median = 11.33 months, 95% CI = 9.8–14.22) (**Figures 3C, D** **)**.

**Table 2:** Cox regression of tumor budding for pancreatic ductal adenocarcinoma.

Variable	Estimate	HR (95% CI)	*p*-value
TB at the tumor front	0.7804	2.1823 (0.9706–4.9070)	0.0591
TB in the tumor center	0.3769	1.4577 (0.6257–3.3961)	0.3824
TB both at the tumor front and in the tumor center	0.4826	1.6202 (0.7828–3.3535)	0.1935
TB count	0.0083	1.0084 (0.9889–1.0283)	0.4035

TB score = 0.7804 × (budding location = PTB) + 0.37688 × (budding location = ITB) + 0.48256 × (budding location = TTB) + 0.00833 × budding count.

HR, hazard ratio; CI, confidence interval; TB, tumor budding.

### Univariate Analysis Between All Variables and OS

**Figure 4:**
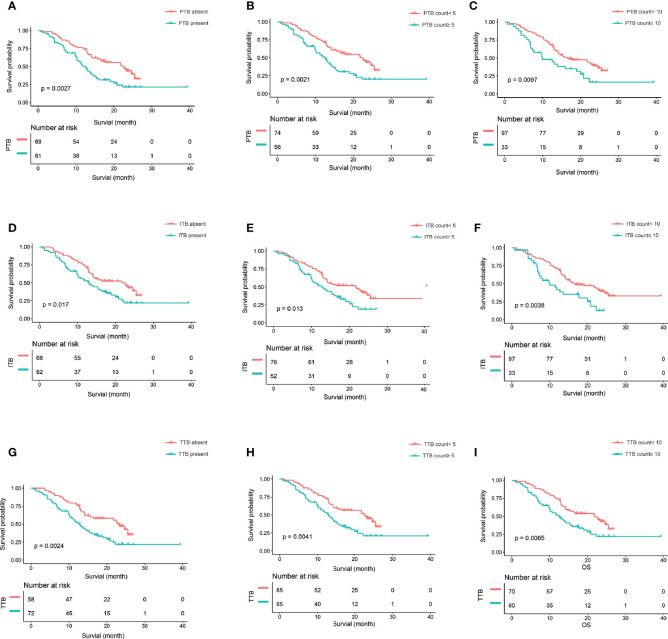
The Kaplan-Meier curve and log-rank test. **(A–C)** The Kaplan-Meier curve and log-rank test suggest that patients in the PTB absent, PTB count<5, and PTB count<10 survived significantly longer than those in the PTB present, PTB count>5, and PTB count>10, respectively. **(D–F)** The Kaplan-Meier curve and log-rank test suggest that patients in the ITB absent, ITB count<5, and ITB count<10 survived significantly longer than those in the ITB present, ITB count>5, and ITB count>10, respectively. **(G–I)** The Kaplan-Meier curve and log-rank test suggest that patients in the TTB absent, TTB count<5, and TTB count<10 survived significantly longer than those in the TTB present, TTB count>5, and TTB count>10, respectively. PTB,, peritumoral budding; ITB, intratumoral budding; TTB, total tumor budding (PTB + ITB).

Sex [hazard ratio (HR) = 0.58, 95% CI = 0.37–0.91, *p* = 0.02], location (HR = 0.58, 95% CI = 0.37–0.92, *p* = 0.02), T category (T2: HR = 2.40, 95% CI = 1.03–5.60, *p* = 0.04), N category (N2: HR = 2.09, 95% CI = 1.22–3.61, *p* = 0.008), and peripancreatic fat invasion (HR = 1.72, 95% CI = 1.07–2.77, *p* = 0.03) were significantly associated with OS (**Table 1**). The TB location, TB count including continuous and categorical variables, and the TB score including continuous and categorial variables were also significantly associated with OS (**Table 3**). The TB score, either as a continuous (*p* = 0.0012) or a categorical (*p* = 0.0003) variable, was more significantly associated with OS in the univariate analysis (**Table 3**) and had a more significantly stratified population (*p* = 0.0002) than did the TB counts (**Figures 3D**, **4**).

**Table 3:** Results of the univariate analysis between tumor budding and overall survival.

Variable	Statistics	HR (95% CI)	*p-*value
TB location, *n* (%)			
No	58 (44.62)	1.0	
PTB	10 (7.69)	2.37 (1.08–5.19)	0.03
ITB	11 (8.46)	1.57 (0.69–3.60)	0.28
TTB	51 (39.23)	2.02 (1.25–3.28)	0.004
PTB count, *n* (median, range)	0 (0–43)	1.03 (1.01–1.05)	0.01
PTB, *n* (%)			
No	69 (53.08)	1.0	
Yes	61 (46.92)	1.93 (1.25–2.99)	0.003
PTB, *n* (%)			
<5	74 (56.92)	1.0	
≥5	56 (43.08)	1.95 (1.26–3.02)	0.003
PTB, *n* (%)			
<10	97 (74.62)	1.0	
≥10	33 (25.38)	1.85 (1.15–2.97)	0.01
ITB count, *n* (median, range)	0 (0–41)	1.03 (1.01–1.05)	0.01
ITB, *n* (%)			
No	68 (52.31)	1.0	
Yes	62 (47.69)	1.69 (1.09–2.62)	0.02
ITB, *n* (%)			
<5	78 (60.00)	1.0	
≥5	52 (40.00)	1.73 (1.12–2.69)	0.01
ITB, *n* (%)			
<10	97 (74.62)	1.0	
≥10	33 (25.38)	2.00 (1.24–3.22)	0.005
TTB count, *n* (median, range)	5.5 (0–72)	1.02 (1.01–1.03)	0.005
TTB, *n* (%)			
No	58 (44.62)	1.0	
Yes	72 (55.38)	1.99 (1.26–3.13)	0.003
TTB, *n* (%)			
<5	65 (50.00)	1.0	
≥5	65 (50.00)	1.89 (1.21–2.94)	0.005
TTB, *n* (%)			
<10	70 (53.85)	1.0	
≥10	60 (46.15)	1.82 (1.17–2.81)	0.007
TB score	0.49 (0.00-1.08)	2.71 (1.48–4.96)	0.0012
TB score, *n* (%)			
TB score-low group	84 (64.62)	1.0	
TB score-high group	46 (35.38)	2.28 (1.46–3.54)	0.0003

HR, hazard ratio; CI, confidence interval; TB, tumor budding; PTB, peritumoral budding; ITB, intratumoral budding; TTB, total tumor budding (PTB+ITB).

### Multivariate Analyses

In the crude model (model 1), the TB location (PTB: HR = 2.37, 95% CI = 1.08–5.19, *p* = 0.03; TTB: HR = 2.02, 95% CI = 1.25–3.28, *p* = 0.004), PTB count (HR = 1.03, 95% CI = 1.01–1.05, *p* = 0.014), ITB count (HR = 1.03, 95% CI = 1.01–1.05, *p* = 0.008), TTB count (HR = 1.02, 95% CI = 1.01–1.03, *p* = 0.005), and the TB score (HR = 2.71, 95% CI = 1.48–4.96, *p* = 0.001) were significantly associated with OS. In the minimally adjusted model 2, the effect size also showed a significant association with OS; this association persisted after applying the fully adjusted model 3, except for the ITB count (HR = 1.02, 95% CI = 1.00–1.04, *p* = 0.06). In addition, on treating the TB score as a categorical variable, the results remained the same. The results of the multivariate analysis are shown in **Table 4**.

**Table 4:** Relationship between the budding and overall survival in different models.

Variable	Model 1		Model 2		Model 3	
	HR (95% CI)	*p-*value	HR (95% CI)	*p-*value	HR (95% CI)	*p-*value
TB location						
No	1.0		1.0		1.0	
PTB	2.37 (1.08–5.19)	0.03	2.30 (1.02–5.18)	0.04	2.39 (1.05–5.44)	0.04
ITB	1.57 (0.69–3.60)	0.28	1.41 (0.61–3.23)	0.42	1.28 (0.55–2.96)	0.57
TTB	2.02 (1.25–3.28)	0.004	1.92 (1.17–3.15)	0.01	1.78 (1.09–2.92)	0.02
PTB count	1.03 (1.01–1.05)	0.014	1.03 (1.01–1.05)	0.009	1.02 (1.00–1.05)	0.03
ITB count	1.03 (1.01–1.05)	0.008	1.03 (1.01–1.05)	0.010	1.02 (1.00–1.04)	0.06
TTB count	1.02 (1.01–1.03)	0.005	1.02 (1.01–1.03)	0.005	1.01 (1.00–1.02)	0.03
TB score	2.71 (1.48–4.96)	0.001	2.64 (1.40–4.95)	0.003	2.35 (1.27–4.33)	0.006
TB score						
TB score-low group	1.0		1.0		1.0	
TB score-high group	2.28 (1.46–3.54)	0.0002	2.15 (1.37–3.38)	0.0009	2.06 (1.32–3.22)	0.0015

Model 1: adjusted for other covariates; model 2: adjusted for age, sex, and body mass index; model 3: further adjusted for location, T category, N category, and peripancreatic fat infiltration.

HR, hazard ratio; CI, confidence interval; TB, tumor budding; PTB, peritumoral budding; ITB, intratumoral budding; TTB, total tumor budding (PTB+ITB).

### Univariate Analysis Between All Variables and the TB Score

The results of the univariate analysis are demonstrated in **Table 5**. The grade of differentiation [odds ratio (OR) = 0.27, 95% CI = 0.12–0.41, *p* = 0.0004] was significantly associated with the TB score.

**Table 5:** Results of the univariate analysis between all variables and the tumor budding score.

Variable	OR (95% CI)	*p-*value
Sex		
Male	0	
Female	−0.07 (−0.19 to 0.06)	0.31
Age	0.01 (−0.00 to 0.01)	0.06
BMI	0.00 (−0.00 to 0.01)	0.46
Tumor size	0.01 (−0.03 to 0.06)	0.52
Location		
Head	0	
Body and tail	−0.07 (−0.19 to 0.06)	0.31
Grade of differentiation		
Well–moderate	0	
Poor–undifferentiated	0.27 (0.12–0.41)	0.0004
T category		
T1	0	
T2	0.08 (−0.12 to 0.28)	0.44
T3	0.14 (−0.07 to 0.35)	0.20
N category		
N0	0	
N1	−0.01 (−0.17 to 0.15)	0.90
N2	0.11 (−0.04 to 0.26)	0.14
Peripancreatic fat invasion		
No	0	
Yes	0.08 (−0.05 to 0.21)	0.24
Resection margin		
R0	0	
R1	−0.07 (−0.19 to 0.06)	0.30

OR, odds ratio; CI, confidence interval; BMI, body mass index.

## Discussion

In this study, we developed a TB score by combining the budding location and the budding counts. To our knowledge, the TB score in PDAC has not been reported in previous studies. Our findings showed that the TB score, either as a continuous (*p* = 0.006) or a categorical (*p* = 0.0015) variable, was more significantly associated with OS in the multivariate analysis than was the PTB counts (0.03) (**Table 4**). We also found that the TB score significantly stratified the population (*p* = 0.00018) and was a little bit better than the PTB counts (cutoff PBT = 5, *p* = 0.0021; cutoff PBT = 10, *p* = 0.0097) (**Figures 3D**, **4B, C**). Therefore, the TB score may be a reproducible and reliable prognostic marker enabling better patient stratification and guiding more successful and individualized therapy.

To date, some studies have evaluated the association between TB and prognosis in patients with PDAC. TB in PDAC was first reported by Karamitopoulou et al. (13), who assessed it using pan-cytokeratin staining. They grouped 117 patients with PDAC into low-grade budding and high-grade budding (high-grade budding was defined as an average of >10 buds across HPFs) and found that high-grade budding was linked to higher pT classification (*p* = 0.0463), lymphatic invasion (*p* = 0.0192), and decreased disease-free survival (*p* = 0.0005) and OS (*p* < 0.0001). O’Connor et al. (15) assessed the TB of 192 patients with PDAC using HE sections and found that the presence of TB was an independent adverse prognostic factor in patients with PDAC. Petrova et al. (17) analyzed the association between TB and perineural invasion and their prognostic role in 119 cases of PDAC using HE sections and found that TB was an independent negative prognostic factor for PDAC. Lawlor et al. (16) classified 613 patients with PDAC into high-grade (*n* = 251) and low-grade (*n* = 362) TB groups and found an increased risk of all-cause mortality [relative risk (RR) = 1.46, 95% CI = 1.13–1.88, *p* = 0.004; HR = 2.65, 95% CI = 1.79–3.91, *p* < 0.0001] and recurrence (RR = 1.61, 95% CI = 1.05–2.47, *p* = 0.03) in PDAC patients with high-grade TB. In this study, we divided the TB counts into two groups according to cutoff points of 5 and 10. We found that the PTB counts (*p* = 0.03) and location (*p* = 0.04) were significantly associated with OS in the multivariate analysis after adjusting for other significant variables. This result was consistent with the findings of previous reports.

However, previous reports have only focused on the “classic” PTB, and ITB has been reported in only a few studies. Lugli et al. (18) found that high-grade ITB was a poor prognostic factor in univariate (*p* = 0.001) and multivariate (*p* = 0.019) analyses on adjusting for T category, N category, distant metastasis, and adjuvant therapy in patients with colorectal cancer. Another study showed that complete pathological response was independently and significantly associated with a defective–mismatch repair system (OR = 2.61, 95% CI = 1.355–5.040, *p* = 0.004) and a low degree of ITB (OR = 2.52, 95% CI = 1.366–4.894, *p* = 0.025) (19). The results of a study by Marx et al. (20) showed that ITB was a prognostic biomarker in stage II colorectal cancer. They suggested that this could be the basis to identify patients who might benefit from adjuvant therapy, especially those in whom PTB was difficult to assess. In this study, we found that the ITB location (HR = 1.28, 95% CI = 0.55–2.96, *p* = 0.57) and ITB counts (HR = 1.02, 95% CI = 1.00–1.04, *p* = 0.06) were not significantly associated with OS in the multivariate regression models after adjusting for other significant variables. However, the TB location had an effect on the results. We found that the PTB (*p* = 0.04) and TTB (*p* = 0.02) locations were significantly associated with OS in the multivariate regression models after adjusting for other significant variables. Therefore, we developed a combined TB score based on the TB location (peritumor and intratumor) and the TB counts (PTB+ITB). We found that the TB score, either as a continuous (*p* = 0.006) or a categorical (*p* = 0.0015) variable was more significantly associated with OS in the multivariate analysis and had a more significantly stratified population (*p* = 0.0002) than did the PTB counts (cutoff PBT = 5, *p* = 0.0021; cutoff PBT = 10, *p* = 0.0097).

Clinical issues can be resolved using digital pathology (26, 27). Rendering routine pathological diagnoses using whole slide imaging is a feasible approach. Several studies (28, 29) have compared diagnostic concordance between using digital slides and using conventional glass slides, and the results showed a range of concordance from 89% to 99%. In this study, we not only used digital whole slide imaging but also made all specimens into large sections (area = 76 mm × 52 mm). Whole slide pathology images of large sections have some advantages. Firstly, pancreatic tumor and peripheral normal pancreatic tissue can be fully displayed in the same slide. Furthermore, the pathologists can accurately locate the hotspot and evaluate the peritumoral and intratumoral budding by manual drawing.

### Limitations

Our study has some limitations. Firstly, this study was retrospective and based on data obtained from a single center. Secondly, the sample size was small. Thirdly, TB has been proven to be associated with EMT in cancer. However, we did not explore the association between TB and EMT in this study. Fourthly, we did not use pan-cytokeratin staining to visualize the TB more clearly in sections. Fifthly, we did not compare the results between whole slide pathology images of large sections and those of a traditional microscope. However, the specimens of patients in this study were made into large sections (area = 76 mm × 52 mm) and not into traditional small sections (area = 76 mm × 26 mm). Finally, although we found that the TB score significantly stratified population (*p* = 0.0002), significant superiority to PTB counts was not found. Future studies need to focus on multicenter validation with a larger sample size to obtain high-level evidence for the clinical application of the TB score. The use of the digital whole slide image in the pathologic diagnosis should also be promoted.

## Conclusions

The TB score is strongly and independently associated with the risk of OS in patients with PDAC. Thus, it can be used as a novel prognostic indicator and can guide individualized therapeutic approaches for patients with PDAC in the future.

## Data Availability Statement

The original contributions presented in the study are included in the article/supplementary material. Further inquiries can be directed to the corresponding authors.

## Ethics Statement

The studies involving human participants were reviewed and approved by Changhai Hospital. Written informed consent for participation was not required for this study in accordance with the national legislation and the institutional requirements.

## Author Contributions

YB conceptualized the study, performed validation and formal analysis, curated the data, and prepared the original draft. HJ contributed to the study methodology, supervised the study, and administered the project. YQ was responsible for the software. YY helped with investigation. HJ and YB helped with resources. CS and JL reviewed and edited the paper. JZ contributed to visualization. YB, JL, and HJ helped with funding acquisition. All authors contributed to the article and approved the submitted version.

## Funding

This work was supported in part by the National Science Foundation for Scientists of China (81871352, 82003107, 82171915, and 82171930), Clinical Research Plan of SHDC (SHDC2020CR4073), 234 Platform Discipline Consolidation Foundation Project (2019YPT001, 2020YPT001), and The Natural Science Foundation of Shanghai Science and Technology Innovation Action Plan (20ZR1456800, 21ZR1478500, and 21Y11910300).

## Conflict of Interest

The authors declare that the research was conducted in the absence of any commercial or financial relationships that could be construed as a potential conflict of interest.

## Publisher’s Note

All claims expressed in this article are solely those of the authors and do not necessarily represent those of their affiliated organizations, or those of the publisher, the editors and the reviewers. Any product that may be evaluated in this article, or claim that may be made by its manufacturer, is not guaranteed or endorsed by the publisher.
